# The "grey area" of the transfusion practice in the intensive care unit

**DOI:** 10.1186/2197-425X-3-S1-A917

**Published:** 2015-10-01

**Authors:** FJ Kranenburg, MS Arbous, S Le Cessie, JG Van der Bom

**Affiliations:** Sanquin Research, Transfusion Medicine, Leiden, Netherlands; Leiden University Medical Center, Clinical Epidemiology, Leiden, Netherlands; Leiden University Medical Center, Intensive Care Unit, Leiden, Netherlands; Leiden University Medical Center, Medical Statistics and Bioinformatics, Leiden, Netherlands; Jon J van Rood Center for Clinical Transfusion Research, Leiden, Netherlands

## Introduction

Present-day transfusion protocols typically specify haemoglobin (Hb) levels as transfusion triggers (i.e. patients below a specified Hb level are almost universally transfused with red blood cells (RBCs), patients above a second threshold are rarely transfused). Between these two Hb levels lies a range (the so called 'grey area') in which RBC transfusion practices differ based on estimated susceptibility of patients to hypoxic complications.

## Objectives

We aimed to quantify the Hb levels determining the boundaries of this grey area within a critically ill ICU population.

## Methods

We conducted an observational cohort study at the combined medical-surgical intensive care unit (ICU) of the Leiden University Medical Center (LUMC) using routinely collected data from previously admitted critically ill patients. Our study cohort consists of all adult, critically ill patients developing moderate to severe anaemia (Hb ≤10 g/dL) at any point during their admission to the ICU between November 2004 and October 2014. We assessed the proportion of Hb measurements resulting in RBC transfusion within 6 hours for the various Hb strata.

## Results

Our cohort consisted of 9,760 patients representing 11,306 admissions on which 127,558 hemoglobin measurements were performed. In total 11.1% of the collected Hb measurements preceded a RBC transfusion by 6 hours or less. Table 1 shows the proportions of Hb measurements leading to transfusion within 6 hours stratified by Hb-level.

**Table 1:** Hb measurements leading to RBC transfusion.

Hb levels (g/dL)	No RBC transfusion within 6 hours after Hb measurement	RBC transfusion within 6 hours after Hb measurement	Total No. of Hb measurements
<5	182 (63.2%)	106 (36.8%)	288
5 - 6	285 (44.5%)	355 (55.5%)	640
6 - 7	1,634 (52.0%)	1,508 (48.0%)	3,142
7 - 8	12,651 (68.0%)	5,899 (32.0%)	18,460
8 - 9	36,324 (91.4%)	3,395 (8.6%)	39,719
9 - 10	34,862 (94.6%)	2,004 (5.4%)	36,866
> 10	27,500 (96.7%)	943 (3.3%)	28,443
*Total*	113,348 (88.9%)	14,210 (11.1%)	127,558

## Conclusions

Our results suggest the 'grey area' to have an upper Hb boundary of around 8 g/dL. However a clear lower boundary cannot be elucidated from this data set as we did not observe the increase in incidence of RBC transfusion we expected to see with extremely low Hb levels. Possible explanations are that this extremely low Hb stratum encompassed patients who were Jehovah's witnesses, patients with other restrictions/limitations to receive transfusion, or patients in which life-sustaining treatment was withheld. The lack of uniform RBC transfusion triggers thus stems from clinical factors, considered by the treating physician, other than hemoglobin levels. This finding is consistent with most transfusion guidelines, which indeed advise the taking of other clinical factors into account when making decisions regarding RBC transfusion.

